# Vascular hyperreactivity in black Cameroonian hypertensive and normotensive patients: a comparative study

**DOI:** 10.11604/pamj.2017.28.2.13471

**Published:** 2017-09-04

**Authors:** Sylvie Ndongo Amougou, Dieudonné Danwe, Hamadou Ba, Bonaventure Jemea, Liliane Kuate Mfeukeu, Christian Ngongang Ouankou, Jingi Musa Ahmadou, Samuel Kingue

**Affiliations:** 1 Department of Internal Medicine, Faculty of Medicine and Biomedical Sciences, University of Yaoundé I, Yaoundé, Cameroon; 2 University Teaching Hospital Yaoundé, Yaoundé, Cameroun; 3 Yaounde Central Hospital, Yaoundé, Cameroun; 4 Department of Surgery and Specialties, Faculty of Medicine and Biomedical Sciences, University of Yaoundé I, Yaoundé, Cameroon; 5 Yaoundé General Hospital, Yaoundé, Cameroun

**Keywords:** Vascular hyperreactivity, high blood pressure, black Cameroonian, cold test

## Abstract

**Introduction:**

Vascular hyperreactivity is a risk factor and a factor predicting hypertension (high blood pressure). Unlike other continents where several studies were carried out, it has rarely been studied in black Africa in general and in Cameroon in particular.

**Methods:**

Vascular reactivity was measured by the cold test. Vascular hyperreactivity was defined as an increase in blood pressure > 20 mmHg for systolic and/or > 15 mmHg for diastolic. Khi^2^, Man-Withney, Wilcoxon's signed ranks and logistic regression tests were used for statistical analysis.

**Results:**

A total of 31 hypertensive and 31 normotensive patients matched by age and sex participated in this study. Vascular hyperreactivity was present in 77.4% hypertensive patients and 51.6% normotensive patients. There was a significant association between vascular hyperreactivity and hypertension [OR = 3.2 (1.07 - 9.63), p = 0.034]. The median arterial pressure was higher in responders compared to non-responders in the normotensive group. Age > 45 years, female sex, obesity and family history of hypertension appeared to be associated with vascular hyperreactivity, but only in normotensive patients.

**Conclusion:**

Vascular hyperreactivity appears to be a risk factor for high blood pressure in black Cameroonians. It appeared to be associated with low blood pressure, age, sex, obesity and family history of hypertension but this was only in the normotensive.

## Introduction

High blood pressure (HBP) is defined by the World Health Organization (WHO) as a systolic blood pressure (SBP) above or equal to 140 mmHg and/or diastolic blood pressure (DPB) above or equal to 90 mmHg. HBP is a major public health problem in the world. According to the WHO, the estimated average prevalence of hypertension in 2008 among adults aged 25 and over was 40%, the highest rate being recorded in Africa (46%). According to Kearney et al, 79 million people in sub-Saharan Africa were hypertensive in 2000 and there would be twice as many people affected by 2025 [1]. In Cameroon, Kingue et al in 2015 recorded a prevalence of 29.7% [2]. Cardiovascular diseases are the leading cause of death in the world according to the WHO. The latter estimated the number of HBP-related deaths in 2015 to be 15 million [3]. There are two main types of hypertension: essential hypertension for which no direct cause has been identified representing 95% of cases and secondary hypertension (‹ 10% of cases) of which cause is usually found. In essential hypertension, when there is no specific cause, risk factors predisposing individuals to the development of hypertension are identified. Vascular hyperreactivity to vasopressor agents-noradrenaline, serotonin, vasopressin and angiotensin II-is one of these risk factors. This hyperreactivity would be a predictive factor in the further development of an essential hypertension and is potentiated in hypertensive people due to arterial remodeling in response to the permanent rise in blood pressure [4, 5]. In 1979, Wood et al found in the United States of America that 71% of the "hyperreactors" had developed an essential hypertension, compared with only 19% of the "normoreactors" after a follow-up period of 45 years. Several authors reached the same conclusion [6, 7] but others found contradictory results [8, 9]. In addition, vascular reactivity would be higher in black than in Caucasians [10]. Vascular hyperreactivity has been identified as a risk factor in many studies conducted in other continents than Africa. Indeed, very few studies were conducted in Africa in general and in Cameroon in particular with the aim of determining the place of the vascular hyperreactivity in the genesis of hypertension. We performed this study in order to compare the vascular hyperreactivity of a group of normotensive subjects and that of a group of subjects with essential arterial hypertension.

## Methods

The study was carried out at the Yaoundé Teaching Hospital (CHUY). It was approved by the Institutional Committee for Research Ethics (CIER) of the Faculty of Medicine and Biomedical Sciences (FMSB) of the University of Yaounde I. We received research authorization from the CHUY and we obtained informed consent from each participant. In the study we included hypertensive subjects aged 18 to 60 years, not taking antihypertensive drugs and clinically healthy normotensive patients who were matched to the hypertensives by age and sex with a ratio of 1:1. Patients with a history of heart disease, chronic obstructive pulmonary disease, chronic kidney disease and peripheral neuropathy, pregnant women, subjects with grade 3 HA (PA ≥ 180/110) and those with signs of Secondary HTA were excluded. Age, gender and background were recorded during the interview. The weight was measured using an electronic weighing scale with an accuracy of 100 grams (OMRON^®^ HN289) and the size was measured with a gauge to the nearest centimeter. Body mass index (BMI) was calculated by making the weight ratio on the square of the height. Blood pressure was measured using automatic tensiometers (OMRON^®^ M^2^ Basic and M^3^). We also looked for physical signs that could lead to secondary hypertension. The vascular reactivity was measured by the cold test with cold water. After a rest period of 15 minutes in supine position, BP was measured three times with a one-minute interval. The average of these three measures was considered the basic BP. Then the temperature of the water was measured (3-6°C) and the subject was invited to immerse his left hand to the wrist for 60 seconds. BP was measured at 60 seconds of immersion after which the subject could extract his hand. Finally, BP was measured every minute for 4 minutes after hand extraction. Vascular hyperreactivity was defined as an increase in SAD > 20 mm Hg and/or PAD > 15 mm Hg. Statistical analyzes were performed using Statistical Package for Social Sciences (SPSS) version 20.0. Khi^2^, Fisher and logistic regression tests were used to measure the association between qualitative variables. The Pearson correlation was used to measure the association between the quantitative variables. The Man-Whitney and Wilcoxon signed rows tests were used for median comparison. The threshold of statistical significance was p < 0.05.

## Results

**Figure 1:**
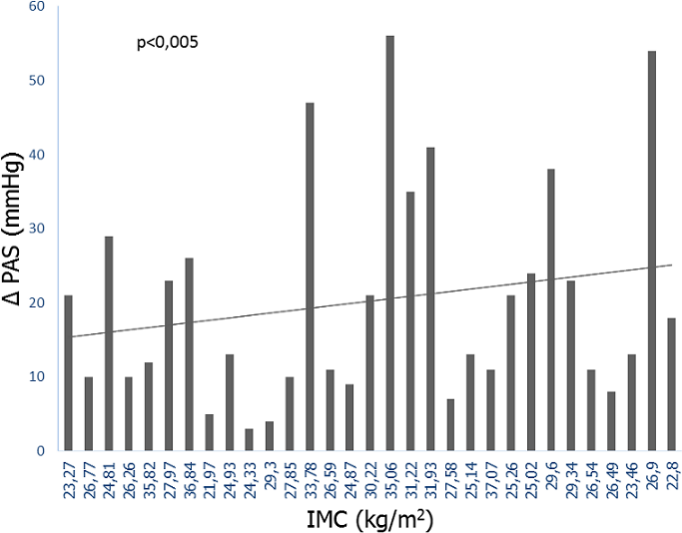
Correlation between systolic reactivity and the body mass index in normotensive

All 31 hypertensive subjects and 31 normotensive subjects participated in this study. Table 1 shows the basic clinical characteristics of hypertensive and normotensive patients. Weight, BMI, SBP, DBP, MBP and the FC were significantly higher in hypertensive compared to normotensive. The magnitude of increase in BP during the cold test was higher in hypertensive patients (Table 2). Of the hypertensive patients, 77.4% had vascular hyperreactivity versus 51.6% in normotensive patients. There was a positive correlation (r = 0.40, p = 0.03) between systolic reactivity and BMI in normotensive patients (Figure 1) but no correlation (r = 0.07, p = 0.72) in hypertensive patients. The medians of BP in resting and BMI (SBP = 119 mm Hg versus 116 mm Hg, DPB = 76 mm Hg versus 74 mm Hg, BMI = 28.66 kg / m^2^ against 26.54 kg / m^2^; P < 0.05) were higher in responders compared to non-responders in the normotensive group. Age > 45 years (1.15 (0.20 - 6.71); P = 0.87), female sex (3.07 (0.47 - 20.05); P = 0.24), obesity (1.90 (0.23 - 15.71); P = 0.55) and family history of HBP (3.73 (0.70 - 19.99); P = 0.12) appeared to be associated with vascular hyperreactivity in normotensive patients. This was not the case in hypertensive patients. There was a significant association between vascular hyperreactivity and hypertension ( (3.21 (1.07 - 9.63); P = 0.03).

**Table 1:** Baseline clinical features of the hypertensive (n=31) and of the normotensive patients (n=31)

	Hypertensive Μ ± σ	Normotensive M ± σ	*P*
Age (years)	45 ± 9	42 ± 9	0.231
Weight (kg)	83.3 ± 11.8	77.0 ± 12.1	0.037
Height (cm)	166 ± 5	166 ± 8	0.849
BMI (kg/m^2^)	30.39 ± 4.19	28.03 ± 4.19	0.027
SBP (mmHg)	142 ± 13	118 ± 9	< 0.001
DBP (mmHg)	91 ± 9	76 ± 7	< 0.001
ABP (mmHg)	108 ± 10	90 ± 7	< 0.001
HR (bpm)	73 ± 11	67 ± 12	0.033

M: median σ: standard deviation BMI : Body Mass IndexSBP: Systolic Blood Pressure DPB: Diastolic Blood Pressure ABP: average Blood Pressure HR: Heart rate

**Table 2:** Average increase of the blood pressure and heart rate during the cold test in hypertensive (n=31) and normotensive patients (n=31)

	Hypertensive M ± σ	Normotensive M ± σ	*P*
SBP (mmHg)	29 ± 14	20 ± 14	0.009
DBP (mmHg)	17 ± 10	14 ± 9	0.141
ABP (mmHg)	21 ± 10	16 ± 10	0.039
HR (bpm)	5 ± 7	5 ± 9	0.622

M: median σ: standard deviation BMI: Body Mass Index SBP: Systolic Blood Pressure DPB: diastolic blood pressure ABP: average Blood Pressure HR: Heart rate

## Discussion

Explaining the fact that the race, the average age of participants in our study was 45 ± 9 years and 42 ± 9 years, respectively, in hypertensive and normotensive patients, is consistent with Benetos and Safar in France in 1991 and Elias et al in Nigeria in 2015 [8, 11]. As expected, SBP, DBP and MBP as well as heart rate were significantly higher in hypertensive than normotensive as reported by other authors [8, 11, 12]. In this study, weight and BMI were also significantly higher in hypertensive patients than in normotensive patients. This is consistent with the results of Elias et al in Nigeria but is contrary to those of Benetos and Safar and Laflèche et al in France [8, 11, 12]. This could be explained by the fact that the prevalence of hypertension and obesity is higher in Africa than in Europe and by the geographical superposition of these two conditions. Kingue et al found a prevalence of obesity of 23.5% in Cameroon in 2015, while Matta et al found a prevalence of obesity of 15.7% in France in 2016 [2, 13]. In our study, SBP, DBP and HR increased significantly during the cold test. The amplitude of increase in SBP and DBP but not in HR was higher in hypertensive than in normotensive corroborating the data in the literature [8, 11]. However, the amplitude of increase in blood pressure and HR during the cold test was higher in our population compared with those obtained by Drummond in Australia, Benetos and Safar and Laflèche et al in France [8, 12 , 14]. A more pronounced hyperactivity of the sympathetic system and a more marked vascular reactivity in the cold test in black may explain these results [10, 15]. We found that 77.4% of hypertensive patients had vascular hyperreactivity compared to 51.6% of normotensive patients. This is consistent with Hagbe et al's 1994 results in Cameroon, which found 72% vascular hyperreactivity in hypertensive patients versus 50% in normotensive patients [16] and shows that vascular reactivity to the cold test is a stable phenomenon within the same population.

Benetos and Safar found lower proportions (64% and 47% respectively in hypertensive and normotensive) despite lower thresholds of hyperreactivity (16 mmHg for SBP and 12 mmHg for DBP). This shows once again that black people are more reactive to the cold test compared to Caucasian and black race is a risk factor for hypertension [8, 15]. BMI was positively correlated with systolic reactivity in normotensive patients but not in hypertensive patients. This suggests that hyperactivity of the sympathetic system, endothelial dysfunction and oxidative stress related to overweight and obesity no longer influence vascular reactivity in hypertensive patients. In our study, we found a significant difference between age > 45, female gender, obesity and family history of HBP only in the normotensive group. None of these factors was associated with vascular hyperreactivity in hypertensive patients. Similarly, Benetos and Safar as well as Rajashekar et al found no association between the family history of HBP and vascular reactivity to the cold test [8, 17]. However, Cahloun et al found that black people with more pronounced vascular hyperreactivity in the cold test were those with a family history of hypertension. This explains why we have an association, even if it is not statistically significant between the family history of hypertension and vascular hyperreactivity in normotensive patients. As for the other factors, Zhang et al found that high age (> 45 years), female sex and elevated BP while resting were factors associated with higher vascular reactivity in the cold test [18]. The tendency to vascular hyperreactivity observed in our obese normotensive patients is explained by hyperactivity of the sympathetic system in overweight or obese people [19]. In this study, the data were insufficient to evaluate the association between smoking, alcohol consumption and vascular reactivity. However, according to Zhang et al, inactivity, overweight and alcohol consumption are associated with vascular hyperreactivity [18]. We found a ratio of 3.2 in our study. Similarly, Hagbe et al found an odds ratio of 2.7 [16]. Wood et al found a relative risk of 3.7 [5]. Differences in the methodology could explain these differences. Vascular hyperresponsiveness to the cold test would therefore represent a risk factor for hypertension.

## Conclusion

Vascular hyperreactivity appears to be a risk factor for high blood pressure in Cameroonian black people. It could depend on the baseline status of the vascular system, age, female gender, obesity and family history of hypertension but only in normotensive patients. This suggests that these factors play an important role in the early stages of development of hypertension and that their influence becomes negligible when it becomes manifest.

### What is known about this topic

- Vascular hyperreactivity was identified as a risk factor in many studies conducted in other continents than Africa.

### What this study adds

- Vascular hyperreactivity appears to be a risk factor for high blood pressure in Cameroonian black people.

## Competing interests

The authors declare no competing interest.
